# Greater Outflow Facility Increase After Targeted Trabecular Bypass in Angiographically Determined Low-Flow Regions

**DOI:** 10.1016/j.ogla.2023.06.008

**Published:** 2023-06-20

**Authors:** Clemens A. Strohmaier, Daniel Wanderer, Xiaowei Zhang, Devansh Agarwal, Christopher B. Toomey, Karl Wahlin, Hao F. Zhang, W. Daniel Stamer, Robert N. Weinreb, Fiona S. McDonnell, Alex S. Huang

**Affiliations:** 1 Department of Ophthalmology and Optometry, Kepler University Hospital, Johannes Kepler University, Linz, Austria.; 2 The Viterbi Family Department of Ophthalmology, Hamilton Glaucoma Center, Shiley Eye Institute, University of California, San Diego, California.; 3 Department of Biomedical Engineering, Northwestern University, Evanston, Illinois.; 4 Department of Biomedical Engineering, Duke University, Durham, North Carolina.; 5 Moran Eye Center, University of Utah, Salt Lake City, Utah.

**Keywords:** Conventional outflow, Minimally invasive glaucoma surgery, Outflow facility, Aqueous humor angiography, Segmental outflow

## Abstract

**Purpose:**

To investigate the impact of trabecular bypass surgery targeted to angiographically determined high- vs. low-aqueous humor outflow areas on outflow facility (C) and intraocular pressure (IOP).

**Design:**

Ex vivo comparative study.

**Subjects:**

Postmortem ex vivo porcine and human eyes.

**Methods:**

Porcine (n = 14) and human (n = 13) whole globes were acquired. In both species, anterior segments were dissected, mounted onto a perfusion chamber, and perfused using Dulbecco’s phosphate buffered solution containing glucose in a constant flow paradigm to achieve a stable baseline. Fluorescein was perfused into the anterior chamber and used to identify baseline segmental high- and low-flow regions of the conventional outflow pathways. The anterior segments were divided into 2 groups, and a 5 mm needle goniotomy was performed in either a high- or low-flow area. Subsequently, C and IOP were quantitatively reassessed and compared between surgery in baseline “high-flow” and “low-flow” region eyes followed by indocyanine green angiography.

**Main Outcome Measures:**

Outflow facility.

**Results:**

In all eyes, high- and low-flow segments could be identified. Performing a 5-mm goniotomy increased outflow facility to a variable extent depending on baseline flow status. In the porcine high-flow group, C increased from 0.31 ± 0.09 to 0.39 ± 0.09 μL/mmHg/min (*P* = 0.12). In the porcine low-flow group, C increased from 0.29 ± 0.03 to 0.56 ± 0.10 μL/mmHg/min (*P* < 0.001). In the human high-flow group, C increased from 0.38 ± 0.20 to 0.41 ± 0.20 μL/mmHg/min (*P* = 0.02). In the human low-flow group, C increased from 0.25 ± 0.11 to 0.32 ± 0.11 μL/mmHg/min (<0.001). There was statistically significant greater increase in C for eyes where surgery was targeted to baseline low-flow regions in both porcine (0.07 ± 0.09 vs. 0.27 ± 0.13, *P* = 0.007 μL/mmHg/min, high vs low flow) and human eyes (0.03 ± 0.03 vs. 0.07 ± 0.02, *P* = 0.03 μL/mmHg/min, high vs. low flow).

**Conclusions:**

Targeting surgery to low-flow areas of the trabecular meshwork yields higher overall facility increase and IOP reduction compared to surgery in high-flow areas.

**Financial Disclosure(s):**

Proprietary or commercial disclosure may be found in the Footnotes and Disclosures at the end of this article.

Intraocular pressure (IOP) is the most important risk factor for glaucoma, and IOP lowering is still the mainstay for glaucoma treatment. Aqueous humor outflow (AHO) resistance through the trabecular meshwork (TM) accounts for 50% to 75% of total outflow resistance in the eye^1–3^ and thus is a major determinant of IOP. TM outflow resistance is increased (or outflow facility [C] is decreased) in glaucoma,^4,5^ and bypassing the TM (either by a stent, ablation, or goniotomy-like surgery) is the mechanism of action for many common minimally invasive glaucoma surgical (MIGS) procedures.^6^ The problem is that in multiple (largescale, well-controlled, and randomized) clinical trials, average MIGS IOP reduction (~1–2 mmHg) is limited.^6,7^

Several clinical and experimental studies have demonstrated the segmental nature of AHO. In many species (studies in postmortem eyes: pig, cow, cat, dog, and human; and studies in living organisms: rodent, cat, dog, nonhuman primates, and humans), segmental AHO has been shown via spatial differences in AHO tracer deposition in the TM using perfused microbeads,^8–10^ TM electron microscopic analyses after cationic ferritin perfusion,^11,12^ and after imaging of intracameral soluble tracers observed on the ocular surface using aqueous angiography.^13–20^ Aqueous angiography arose by modifying tools already FDA (US Food and Drug Administration)-approved and commercially available for retinal angiography. Segmental AHO was then confirmed in healthy human volunteers and patients with glaucoma using aqueous angiography during clinically indicated cataract surgery.^21,22^

We have recently shown that angiographically determined high- and low-flow TM of the AHO pathways differ in their local outflow facilities.^23^ This implies that AHO facility should be described as different in different parts of the TM as opposed to as a single numerical value coming from a uniform TM. This also implies that trabecular bypass surgery could have different effects on overall facility (and thereby IOP) based upon its placement in baseline high- or low-flow TM regions. This is consistent with previous studies where differential AHO patterns were seen after trabecular bypass stent placement in high- and low-flow TM in patients with glaucoma.^21^ While different patterns of qualitative AHO improvement were observed (such as faster outflow signal occurrence or new outflow signal emergence in baseline low-flow regions) after stent implantation, conclusions could not be drawn because the study design was underpowered to detect differences in IOP lowering.^22^

Therefore, we designed a preclinical study where we can precisely study the impact of TM bypass surgical placement location on outflow facility. Two species (pig and human) were tested to increase the generalizability of the results. We tested the hypothesis that TM bypass surgery placement in baseline low-flow regions will have a greater impact on C increase and IOP reduction than placement in baseline high-flow regions. Outcomes are designed to inform ophthalmologists where best to perform MIGS as a way to improve outcomes in patients with glaucoma.

## Methods

This study was carried out in accordance with the Declaration of Helsinki and approved by the institutional review boards of University of California, San Diego (see below). Obtaining written informed consent was not applicable. The study was deemed exempt from animal subjects research by the Institutional Review Board.

Porcine eyes (n = 14) were obtained from an abattoir (shipped on blue ice within 24 hours of death) and trimmed of excess tissue upon arrival. After a 2-minute submersion in betadine, the eyes were stored in balanced salt solution at 4°C until used. Human eyes (n = 13) were obtained from the San Diego Eye Bank under institutional review board approval (UCSD #805646) and used immediately after receipt. The donor age, cause of death, death-to-procurement time, and lens status are listed in Table 1. Only eyes without a history of ocular diseases and without prior ocular surgery except for cataract extraction were included in this study.

### Tissue Preparation and Anterior Segment Perfusion Setup

**Figure 1.**
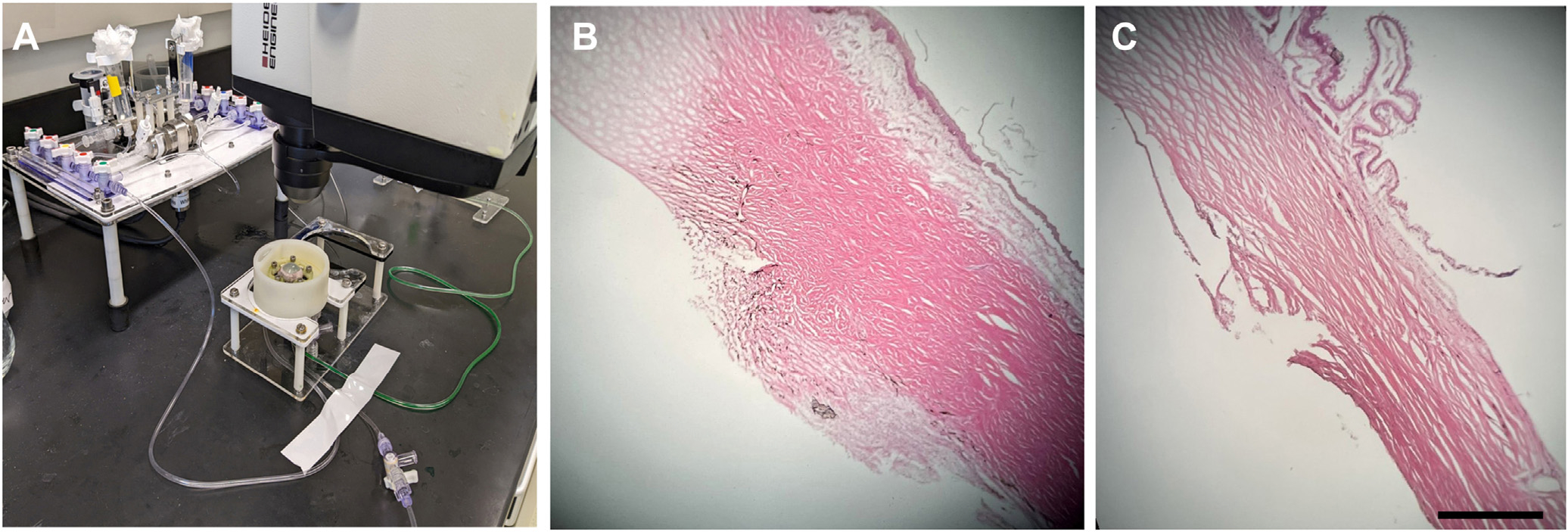
**A,** Experimental setup–Porcine and human anterior segments were perfused using a custom-made system to study outflow facility. **B,** Hematoxylin and eosin staining of the porcine trabecular meshwork after a goniotomy was performed. **C,** Hematoxylin and eosin staining of the human trabecular meshwork after a bent angle needle goniotomy was performed. Scale bar is 500 μm.

**Figure 2.**
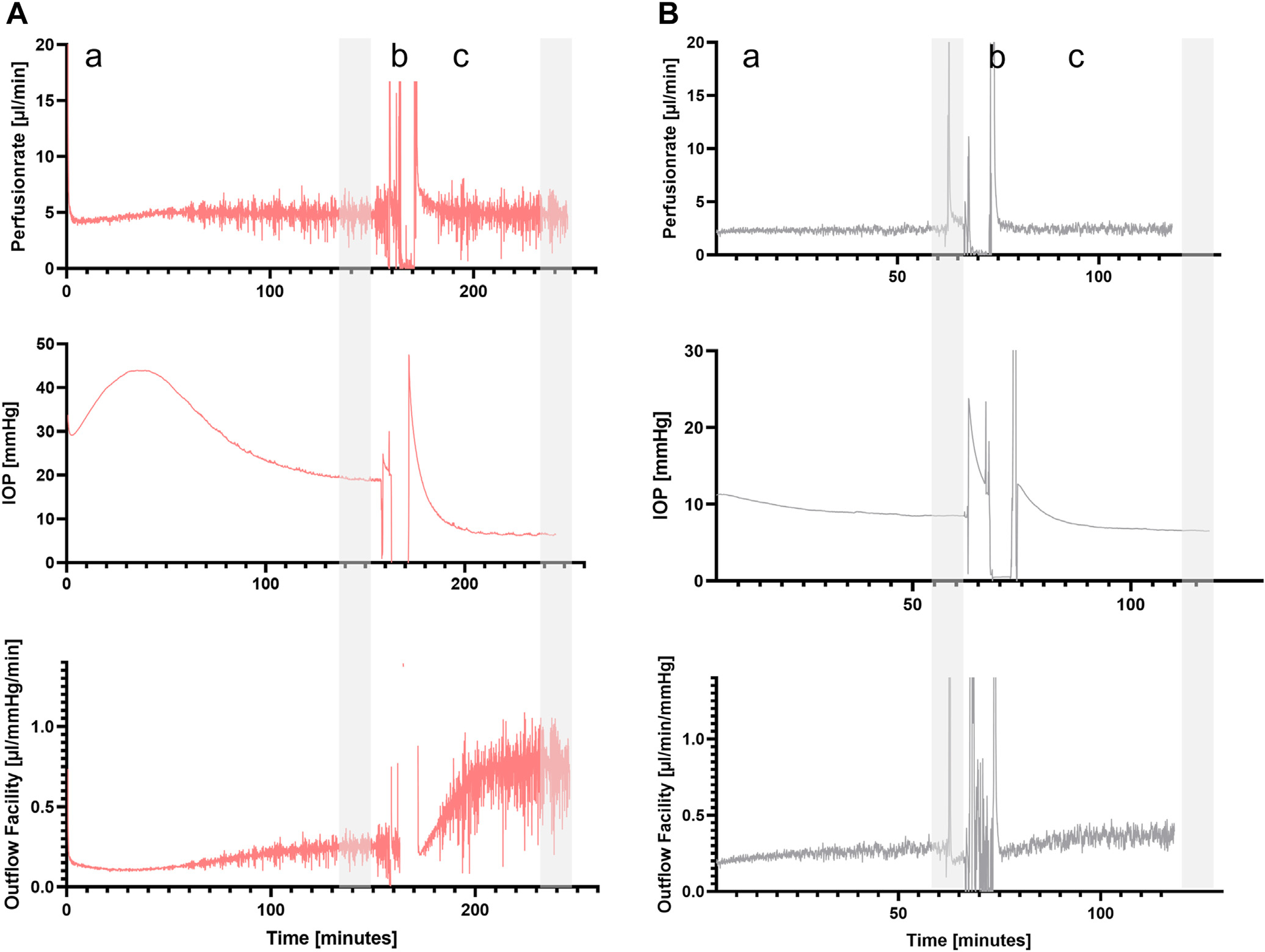
Representative tracings of the experimental protocol. Perfusion rate, IOP, and outflow facility in a porcine anterior segment (**A**, low-flow group) and a human anterior segment (**B**, high flow group) are shown. Each experiment had different phases: (a) initial perfusion until a stable baseline was reached, and (b) unclamping, fluorescein angiography, and targeted goniotomy followed by (c) equilibration phase after goniotomy. For before and after surgery, the vertical gray areas indicate the data segments used for analyses. IOP = intraocular pressure.

The eyes were bisected at the equator to isolate the anterior segment. From the anterior segment, the vitreous, anterior retina, ciliary body, lens, and iris were carefully dissected to expose the TM. Anterior segment preparations were rinsed with balanced salt solution, and any remaining pigment was gently removed with sterile cotton tips. The anterior segments were then mounted onto an anterior segment perfusion chamber at room temperature (Fig 1) (iOnly Human – iPerfusion^24^). The perfusion system includes an anterior segment perfusion chamber connected to a syringe pump (PHD Ultra, Harvard Apparatus) with an interposed flowmeter (SLI-0430, Sensiron AG). The chamber also contained a reference well with liquid at the same height as the limbus of the eye with a pressure transducer (PX409, Omegadyne) placed between the reference well and eye. After the system was filled with sterile-filtered Dulbecco’s phosphate buffered saline containing glucose (1 g/L), the syringe pump was engaged to perfuse the anterior segment at 5 μL/min for porcine eyes and 2.5 μL/min for human eyes. In accordance with previously published studies, only eyes with a baseline facility (calculated as perfusion rate divided by IOP, gray shaded area Fig 2 A[a] and B[a]) between 0.1 – 1.0 μL/mmHg/min (human eyes) and 0.2 – 1.0μL/mmHg/min (porcine) were included in this study.^8,25^

### Experimental Protocol

**Figure 3.**
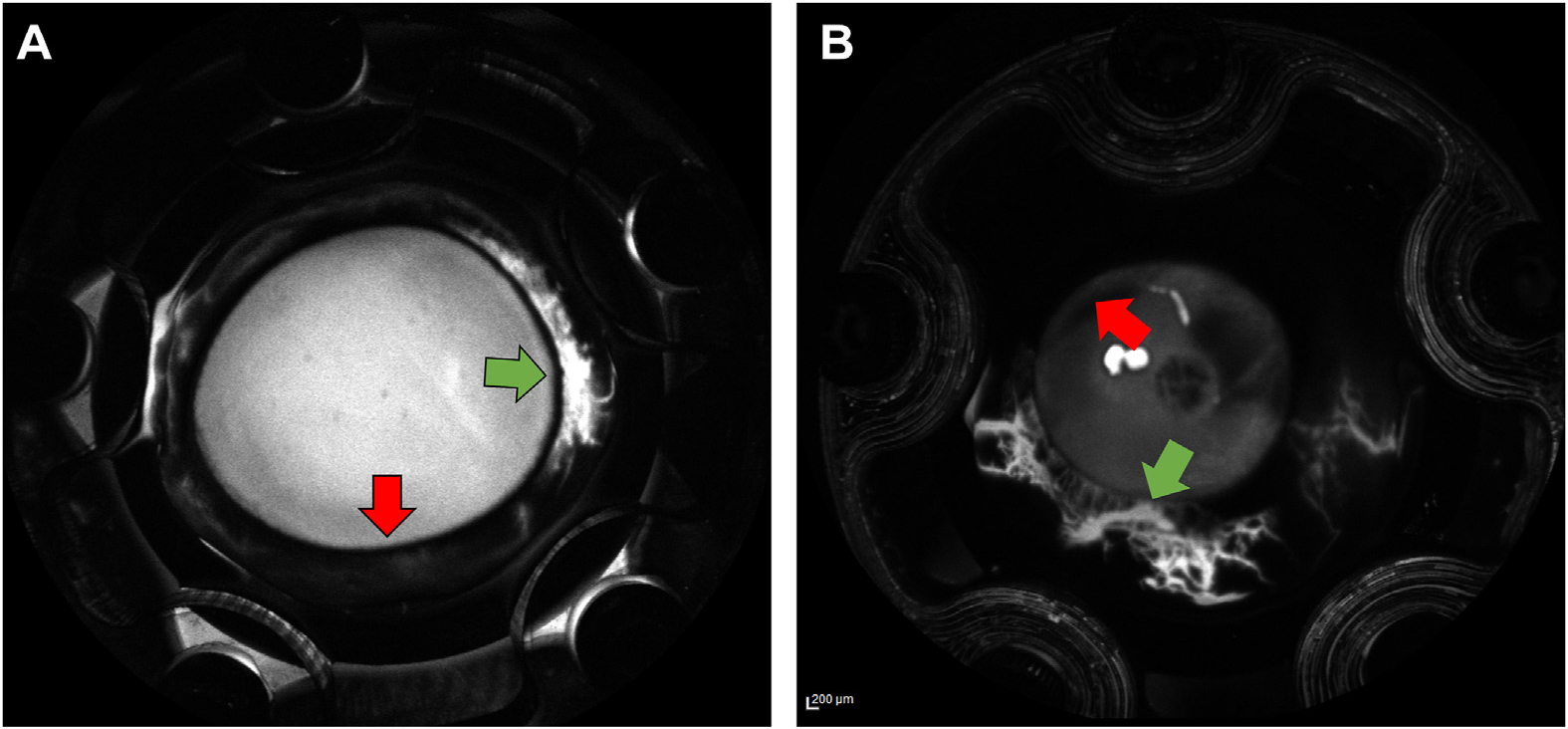
Aqueous humor angiography examples in porcine (**A**) and human (**B**) anterior segments. The images were taken 60 seconds (porcine) and 45 seconds (human) after fluorescein perfusion. In each angiogram, postlimbal high-flow (green arrow) and low-flow (red arrow) regions were identified. Based upon this information, 5 mm segments were selected for goniotomy or bent angle needle goniotomy.

After a stable baseline was achieved (Fig 2; representative tracing),^23^ the anterior segments were perfused with fluorescein (2.5%), with the anterior chamber open to atmospheric pressure. The anterior segments were pressurized again, and AHO images were obtained as described previously.^14,15,22^ In brief, a confocal laser scanning ophthalmoscope (Spectralis FLEX) with built-in filters for fluorescein and indocyanine green (ICG) angiography was pointed perpendicularly at the perfusion chamber holding the anterior segment. Images were taken at set time points of 30 seconds, 60 seconds, 90 seconds, and 2 minutes after tracer introduction for porcine anterior segments. For human anterior segments, 15, 30, 45, and 60 seconds were used as time points. Time points were determined based upon known speed of aqueous angiography signal development from postmortem ex vivo pig and human eyes.^13,22^ In angiographically determined high- or low-flow regions of minimum 5 mm length, the corresponding cornea adjacent to the region was marked, and the eye segment was randomly assigned to either the high- or low-flow experimental group (Fig 3). The distinction between high- and low-flow segments was performed through visual inspection of the angiography image (bright fluorescein signal vs. no fluorescein signal). The 5 mm length was chosen based on prior experiments investigating segmental outflow using AHO angiography.^23^

The eye segments were then unclamped and visualized under a surgical microscope (OMS-90, Topcon Healthcare). For porcine and human anterior segments, the TM was opened for 5 mm using a 25G needle. For porcine eye segments, due to the lack of a continuous Schlemm’s canal, the goniotomy was performed by slicing the TM with the sharp beveled edge of the needle (Fig 1B). For human anterior segments, that contain a true Schlemm’s canal, a bent angled needle goniotomy (BANG) was performed.^26^ BANG is a goniotomy method described and utilized for patient care worldwide.^26^ The beveled tip of the needle was bent, and the angled needle was inserted into Schlemm’s canal. Sliding to the side removed the TM for the marked 5 mm region (Fig 1C).

The eye segments were then reclamped onto the perfusion chamber, and the system was flushed again using Dulbecco’s phosphate buffered saline. Perfusion was continued at 5 μL/min (porcine) or 2.5 μL/min (human). Pressure as well as perfusion rate were continuously measured, and outflow facility was calculated (perfusion rate divided by IOP). Once a new baseline was reached, the system was flushed with ICG (0.4%, Cardiogreen) with the anterior chamber opened to atmospheric pressure. ICG AHO angiography was performed in the same manner as described above for fluorescein.

### Histology

After the perfusion study, some of the anterior segments from both porcine and human eyes were placed in 4% paraformaldehyde in phosphate buffered saline for overnight at 4°C. The anterior segments were then cryoprotected in 30% sucrose dissolved in phosphate buffered saline for 1 day. Wedges, including the TM, were grossly cut under a surgical microscope. These tissue wedges were mounted in optimal cutting temperature compound. Ten-μm-thick cryosections were cut, mounted onto Superfrost Plus slides, dried, taken through ethanol steps (70%–100%) followed by xy-lenes, H-E stained, and mounted with a coverslip. Tissue sections images were captured using a digital camera through a microscope with identical illumination.

### Data Collection and Statistical Analyses

**Figure 4.**
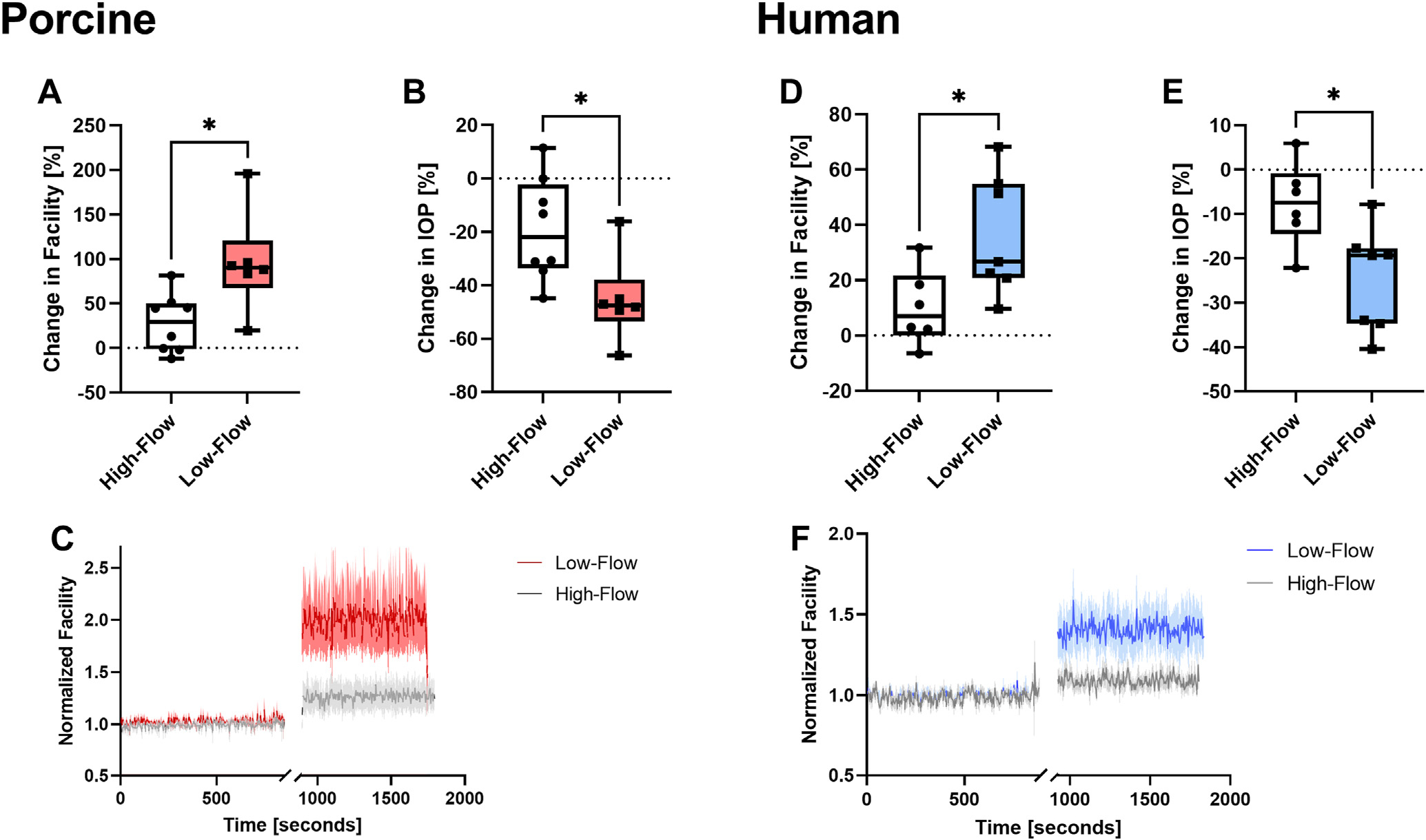
Percentage changes in outflow facility (**A, D**) and IOP (**B, E**) for porcine and human anterior segments. Panels **C** and **F** show the normalized outflow facility and the mean and standard deviation of all analyzed data segments. **A/B** and **D/E** show box and whisker plots with 25%/75% percentiles, median, minimum/maximum values as well as all data points. * denotes *P* < 0.05. IOP = intraocular pressure.

All data were recorded electronically using the iPerfusion software. Data were down-sampled to ~1 Hz and subsequently analyzed in LabChart 8.0. For each anterior segment, 15-minute stretches of the stable baseline conditions were used for statistical analysis. Each anterior segment was normalized to its own baseline, and those normalized values were averaged across all studied anterior segments.^2^ Figure 2 shows an example of the analyzed data segments during the course of the experiment (gray shaded areas). Figure 4 shows the average (and standard deviation) of all normalized data of all experiments. For each anterior segment, the data were normalized to a short 30-second stretch at the beginning of the baseline period. Comparisons were performed using (a) 2-way repeated measures analysis of variance with post hoc *t* tests and Holm–Sidak correction for all measurements obtained in a repeated measures design (i.e., facility) or (b) 2-sided unpaired *t* tests for group comparisons without repeated measures (i.e., relative changes in facility) (Sigmaplot 12.0, Systat Software). A Shapiro–Wilk test was performed to assess normal distribution of the data. Data are presented as mean ± standard deviation, if not stated otherwise. *P* < 0.05 was considered statistically significant.

## Results

Fourteen porcine eyes were used for this study, 8 eyes in the high-flow goniotomy group and 6 eyes in the low-flow goniotomy group. Histology shows successful goniotomy (Fig 1B). Table 2 summarizes the measured outflow facility and IOP values for both groups. Note that these eyes were enucleated and thus have an episcleral venous pressure of zero. Thus, based upon the Goldmann equation, IOP is the perfusion rate divided by the outflow facility, where the perfusion rate was fixed in each species. Baseline outflow facilities in both groups were not statistically significantly different (*P* = 0.62). Fluorescein aqueous angiography was used to determine baseline low- and high-flow regions and to determine the goniotomy site (Fig 3). Porcine goniotomy did not elicit a statistically significant increase in outflow facility in the high-flow group (0.31 ± 0.09–0.39 ± 0.09 μL/mmHg/min, *P* = 0.12), whereas in the low-flow group, it did (0.29 ± 0.03–0.56 ± 0.10 μL/mmHg/min, *P* < 0.001). The change in outflow facility after targeted goniotomy was statistically significant between the 2 groups (High-flow: 0.07 ± 0.09 vs. Low-flow: 0.27 ± 0.13 μL/mmHg/min, *P* = 0.007). Figure 4 summarizes the change in outflow facility relative to baseline (Fig 4A, High-flow: 27.83 ± 32.78% vs. Low-flow: 95.96 ± 56.65%, *P* = 0.02), the change in IOP relative to baseline (Fig 4B, High-flow: −18.98 ± 19.32 vs. Low-flow: −45.31 ± 16.24%, *P* = 0.01), and the normalized outflow facility average and standard deviation of all data segments analyzed (Fig 4C).

For human anterior segment experiments, 13 eyes were used in total: 6 eyes in the high-flow goniotomy group and 7 eyes in the low-flow goniotomy group. Histology shows successful removal of the TM (Fig 1C). Table 3 summarizes the obtained outflow facility and IOP values for both groups. Baseline outflow facilities in both groups were not statistically significantly different (*P* = 0.19). Fluorescein aqueous angiography was used to determine baseline low- and high-flow regions and to determine the site for the BANG (Fig 3). Performing a BANG increased outflow facility in both groups (High-flow: 0.38 ± 0.20–0.41 ± 0.20, *P* = 0.02 and Low-flow: 0.25 ± 0.11–0.32 ± 0.11 μL/mmHg/min, *P* < 0.001). However, the change in outflow facility after targeted BANG was statistically greater in the low-flow group (High-flow: 0.03 ± 0.03 vs. Low-flow: 0.07 ± 0.02 μL/mmHg/min, *P* = 0.03). Figure 4 summarizes the changes in outflow facility relative to baseline (Fig 4D, High-flow: 10.01 ± 13.63% vs. Low-flow: 36.31 ± 21.71%, *P* = 0.03), the change in IOP relative to baseline (4E, High-flow: −7.70 ± 9.44 vs. Low-flow: −24.72 ± 11.71%, *P* = 0.02), and the normalized outflow facility average and standard deviation of all data segments analyzed (Fig 4F).

**Figure 5.**
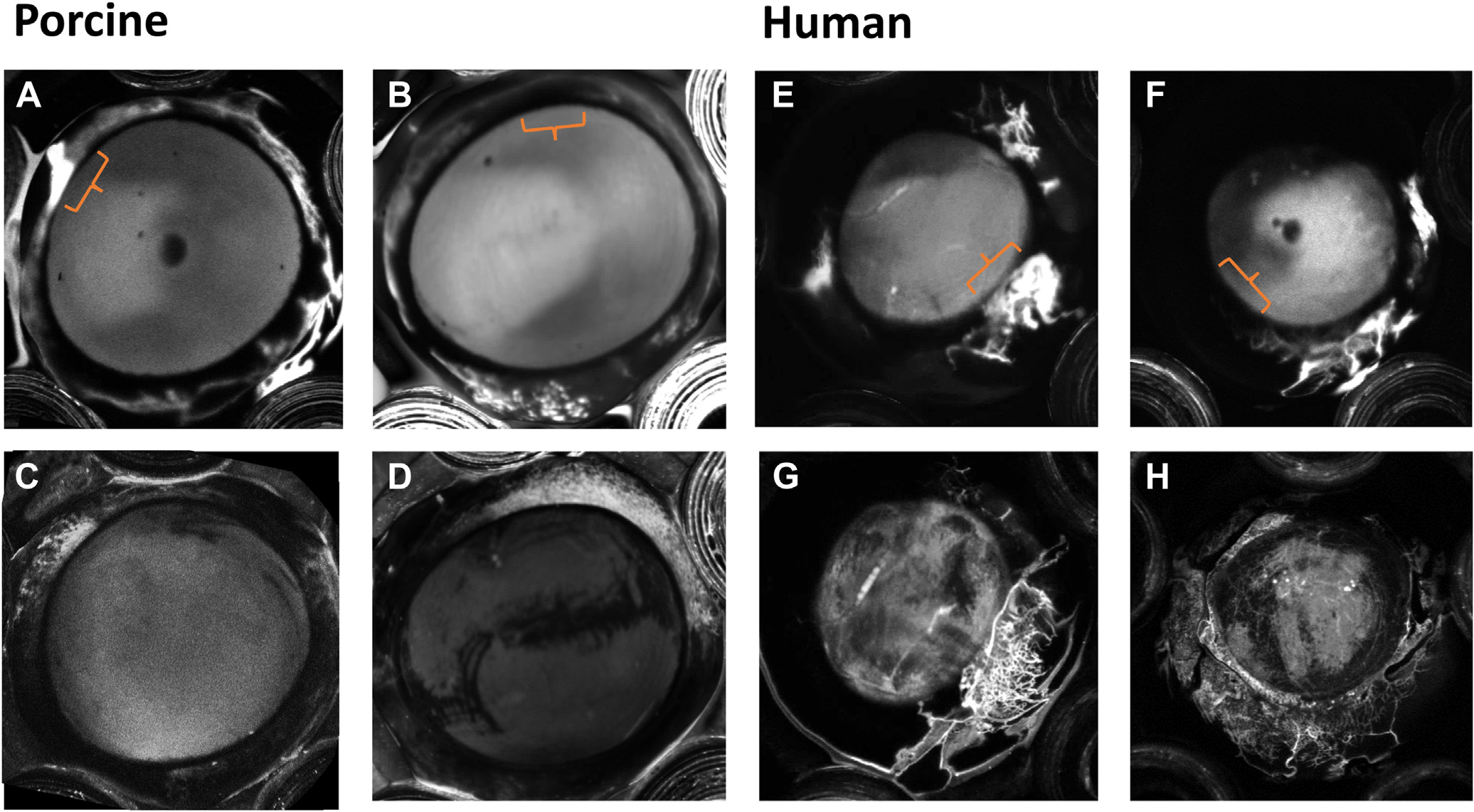
Representative AHO angiography examples of high- and low-flow areas in porcine and human anterior segments. **A, B**, Show fluorescein angiography images taken at 60 seconds after dye injection. As examples, the orange brackets mark the goniotomy site for the high- (**A**) and low-flow (**B**) eyes. **C, D**, Show the corresponding ICG angiography examples taken after the goniotomy was performed (images taken at 2 minutes after dye injection). Panels **E, F, G**, and **H** show the corresponding data for human anterior segments (**E/G** high-flow group and **F/H** low-flow group). AHO = aqueous humor outflow; ICG = indocyanine green.

Figure 5 shows representative examples of fluorescein angiograms (high- and low-flow examples) for porcine (5A, 5B) and human (5C, 5D) high- and low-flow segments being investigated. Panels 5E–5H present the corresponding ICG angiograms after the trabecular bypass surgery was performed.

## Discussion

The presence of segmental AHO is now well established by multiple labs in multiple species using various methods and demonstrated in postmortem experimental eyes and in the eyes of living animals and human subjects^.9,11,14,16,20,27–29^ Thus, in the present study, we tested the hypothesis that targeting (goniotomy-type) TM bypass surgery to baseline low-flow regions would have greater effects on outflow facility than targeting high-flow regions. In order to do so, we studied 2 species (pig and human) using similar trabecular bypass surgical methods, adapted to the specific anatomy of those species. In both porcine and human anterior segment preparations, we found that specifically targeting low-flow regions of the TM yielded relative greater increase in outflow facility and decrease in IOP.

Previous in vivo and in vitro studies did not directly address whether targeting surgery to high- vs. low-flow regions would be more beneficial.^21,22^ Instead, a previous study in postmortem human eyes showed that (stent-based) TM bypass could rescue baseline low-flow regions.^22^ In this laboratory study, facility was not measured, and surgery to high-flow regions was not tested. In patients with glaucoma, we observed similar results, that is, baseline low-flow regions could be rescued after TM bypass.^21^ However, baseline high-flow regions also improved after TM bypass, showing quicker AHO appearance. There were additional challenges. The clinical study was underpowered to detect differences in IOP lowering efficacy. Surgeries were also performed in the nasal side of the eye as current surgical techniques and protocols most commonly direct trabecular bypass stent placement there. Thus, since AHO is generally greater in the nasal region,^30^ it was less common to find a sufficiently large low-flow area big enough to allow 2 stent placements. Thus, to circumvent these challenges, we chose to use an ex vivo model in the present study in order to be able to select TM segments without constraints imposed by surgical access or guidelines.

Regarding the choice of MIGS, needle goniotomy was used in this study due to inherent differences in human vs. porcine anatomy. Porcine eyes do not possess a circular equivalent to Schlemm’s canal and instead have a more tortuous and discontinuous angular aqueous plexus.^31^ Thus, surgical stent implantation may not always access a large lumen. However, this choice of TM bypass surgery limits the available comparative studies using similar procedures. Comparing the present results to the trabecular bypass literature, larger changes in outflow facility have been reported in porcine eye models (using TM ablation), although this could be explained by the larger TM ablations that were performed (~90 degrees, approximately twice the size in our study).^32^ For human eyes, Morton Grant reported that removing 10% to 15% of the TM in 3 eyes resulted in increased outflow facility of 0.07 μL/mmHg/min, which is more consistent with our results.^1^

Toris et al^33^ also recently investigated the effect of current TM bypass devices in a human anterior segment perfusion model. She tested the (a) first generation iStent device, (b) iStent Inject, and (c) the Hydrus microstent. Their average baseline outflow facility was 0.28 μL/mmHg/min (range, 0.22–0.44 μL/mmHg/min) and comparable to our study. The Hydrus microstent had a significantly greater effect on facility compared to the other iStent types, although the Hydrus purports to do more than just bypass the TM. In terms of the “TM only” intervention, the average improvement in outflow facility after iStent was 0.056 μL/mmHg/min. Interestingly, this value is nearly the combined average outflow facility improvement among all human eyes in our study (low- and high-flow conditions combined; 0.05 μL/mmHg/min). Thus, this could indicate that the surgeries by Toris et al were performed in both high- and low-flow regions. Without angiographic imaging, the authors could not know the baseline AHO status of the TM locations where they were performing the surgeries.

**Figure 6.**
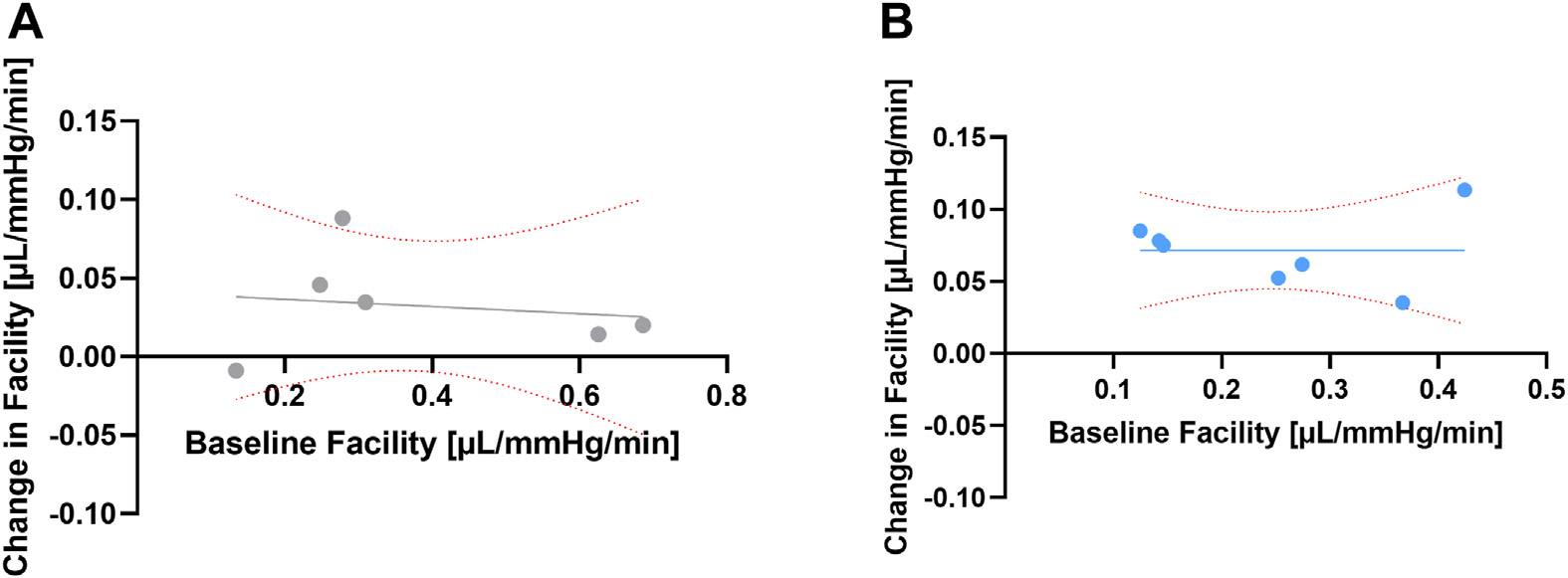
Relationship between baseline facility and change in facility after goniotomy for the high- (**A**) and low-flow (**B**) groups in human anterior segments. The regression lines are not significantly different from zero (*P* = 0.77 and 0.99).

For our experiments with human anterior segments, we point out that baseline outflow facilities between the low- and high-flow surgical eyes were numerically but not statistically different (Table 3). Given that we were employing a constant flow system, this also meant that there was a numerical (but not statistically significant) difference in baseline IOP (Table 3). Previous work had shown that the IOP lowering effects of trabecular microbypass depended on the baseline outflow facility.^34^ Therefore, we tested whether our numerically different (but not statistically significantly different) baseline outflow facility impacted our final results (Fig 6). Comparing baseline outflow facility to facility change using our data showed no such dependency—the slopes of both regression lines were not statistically significantly different from zero. Therefore, this analysis reassures our conclusions and further empathizes with the use of relative change (in percentage change over baseline) to assess outflow facility and IOP.

Limitations are also important to consider in the present study. Anterior segment perfusion models were chosen to simplify the evaluation of targeted surgery on outflow facility and to focus on the TM. However, such a reductionistic approach ignores the contribution of distal outflow biology (Schlemm’s canal and beyond) to total AHO and the clinical response to MIGS. This may explain why TM bypass studies using ex vivo perfusion models can show an ~30% increase in AHO facility compared to more modest IOP reduction (1–2 mmHg compared to cataract surgery alone) in MIGS clinical trials.^6,35^ Therefore, it can be assumed that perfusion models may overestimate the effect on total outflow facility after TM surgery. Thus, the findings of the present study need confirmation in in-vivo studies, both conceptionally as well as regarding the effect sizes obtained. Furthermore, the distal outflow pathways have the capability for active resistance regulation,^36,37^ and neuronal mechanisms governing distal outflow resistance have been identified.^38,39^ The in vivo significance of those mechanisms, however, particularly in response to surgical TM bypass, is currently unknown. Therefore, distal outflow biology and how pairing distal outflow modulation with targeted TM bypass surgery might impact trabecular MIGS efficacy need to be studied.

Lastly, we also point out that human results were of smaller magnitude and with greater variability compared to porcine results (Tables 2 and 3). This may be because animals used in research are genetically more homogenous. All pigs in this study were Yorkshire crossbreed. This is in contrast to human research where genetic diversity is much greater across donor eyes that can be acquired. Furthermore, the anatomical differences in the outflow pathways (as mentioned above) might contribute to the different effect sizes obtained.

In summary, the present study shows an improved effect of trabecular bypass MIGS when they are targeted to baseline low-flow regions. Future clinical studies are needed to confirm this finding in vivo in patients with glaucoma, especially as presumably healthy eyes were used in the present study. The efficacy of TM bypass can be compared with surgery in baseline low- or high-flow regions after angiographic imaging. However, aqueous angiography is laborious, and tools may need to be developed to identify high- and low-flow TM regions during clinical practice more easily. Future research is also needed to search for simpler and ideally noninvasive methods to assess AHO as well.

## Figures and Tables

**Table 1.** Summary of the Cause of Death, Age, Death-to-Procurement Time as Well as Lens Status for All Human Eyes Used

	High-flow Eye Experiments (n = 6)	Low-flow Eye Experiments (n = 7)	

Cause of death (as listed on datasheet)	Interstitial lung disease, sarcoidosis, chronic obstructive pulmonary disease, liver disease, liver cancer	Bladder cancer, prostate cancer, liver cancer, peritoneal cancer, chronic obstructive pulmonary disease, interstitial lung disease	*P* value
Age	63.0 ± 16.0 years	67.86 ± 7.43 years	*P* = 0.49
Death-to-procurement time	7.17 ± 7.54 hours	3.86 ± 0.35 hours	*P* = 0.27
Lens status	5 phakic, 1 pseudophakic	6 phakic, 1 pseudophakic	

**Table 2.** Outflow Facility Values Obtained for Porcine Eyes

	High-flow (n = 8)	Low-flow (n = 6)	Group Comparison (*P* Value)

Baseline facility [μL/mmHg/min]	0.31 ± 0.09	0.29 ± 0.03	0.62
Facility after goniotomy [μL/mmHg/min]	0.39 ± 0.09	0.56 ± 0.10	0.01
Facility change [μL/mmHg/min]	0.07 ± 0.09	0.27 ± 0.13	0.007
*P* value	0.12	<0.001	
Baseline IOP	16.05 ± 3.78	16.56 ± 1.84	0.743
IOP after goniotomy	12.51 ± 2.32	8.82 ± 1.55	0.024
*P* value	0.027	0.003	

IOP = intraocular pressure. *P* values below 0.05 are presented in boldface.

**Table 3.** Outflow Facility Values Obtained for Human Eyes

	High-flow (n = 6)	Low-flow (n = 7)	*P* Value

Baseline facility [μL/mmHg/min]	0.38 ± 0.20	0.25 ± 0.11	0.19
Facility after goniotomy [μL/mmHg/min]	0.41 ± 0.20	0.32 ± 0.11	0.35
Facility change [μL/mmHg/min]	0.03 ± 0.03	0.07 ± 0.02	0.03
*P* value	0.02	<0.001	
Baseline IOP	8.75 ± 4.95	12.41 ± 5.21	0.223
IOP after goniotomy	8.24 ± 5.43	8.81 ± 2.57	0.841
*P* value	0.276	0.018	

IOP = intraocular pressure. *P* values below 0.05 are presented in boldface.

## Abbreviations and Acronyms:

AHO: aqueous humor outflow
ANOVA: analysis of variance
BANG: bent angle needle goniotomy
BSS: balanced salt solution
DPBS: Dulbecco’s phosphate buffered saline
FDA: US Food and Drug Administration
H&E: hematoxylin & eosin
ICG: indocyanine green
IOP: intraocular pressure
IRB: institutional review board
MIGS: minimally invasive glaucoma surgery
PFA: paraformaldehyde
PBS: phosphate buffered saline
O.C.T: Optimal Cutting Temperature Compound
TM: trabecular meshwork
UCSD: University of California San Diego
